# Candida dubliniensis meningitis in an immunocompetent patient: A case report and review of the literature

**DOI:** 10.1016/j.ensci.2024.100519

**Published:** 2024-07-28

**Authors:** Denis Babici, Ali A. Mohamed, Olivia Mattner, Jessica Canosa, Willy Gan, Pooja Patel

**Affiliations:** a Department of Neurology, Florida Atlantic University Charles E. Schmidt College of Medicine, Boca Raton, FL, USA; b Charles E. Schmidt College of Medicine, Florida Atlantic University, Boca Raton, FL, USA

**Keywords:** Case report, Leptomeningeal enhancement, Extracorporeal membrane oxygenation, Injecting drug use, Hepatitis C

## Abstract

**Objective:**

We present the fifth case of candida dubliniensis meningitis in a young immunocompetent host and suggest extracorporeal membrane oxygenation (ECMO) as a potential risk factor for colonization.

**Methods:**

A 22-year-old immunocompetent female presented with a diagnosis of bacterial meningitis. Two years prior, she received ECMO for Covid-19 pneumonia complicated by viral myocarditis & Takutsobo cardiomyopathy. Following discharge, she reported headaches of increasing intensity, all refractory to treatments. Brain magnetic resonance imaging (MRI) was inconclusive. Two weeks prior to her presentation, she was admitted for worsening headaches with cranial nerve VI palsy. Lumbar puncture (LP) revealed white blood cell count (WBC) of 166 cells/μL with neutrophilic predominance and her symptoms progressed, despite 5 days of treatment with broad spectrum antibiotics. All cultures returned negative.

**Results:**

At her current presentation, repeat LP revealed 835 WBC/mm3, 225 mg/dL protein, and 4 mg/100 mL glucose. Brain MRI revealed nodular enhancement in the brainstem and communicating hydrocephalus. MRI of the lumbar spine revealed meningeal enhancement. Cerebrospinal fluid (CSF) cultures came back positive for C.dubliniensis. Treatment began with Amphotericin B and Flucytosine.

**Discussion:**

When clinical suspicion for fungal meningitis is high, repeate LP and CSF analysis is indicated to establish a definitive diagnosis and begin treatment. Additional studies are needed to confirm risk factors, like ECMO, for the colonization of C.dubliniensis, which likely predisposes individuals to invasive candidiasis.

## Highlights

• C. Dubliniensis is a predominantly opportunistic pathogen in immunocompromised host.
• ECMO may be a risk factor for C. dubliniensis colonization in immunocompetent hosts.
• Repeat LP and CSF is indicated to diagnosis C. dubliniensis in immunocompetent host.
• Injecting drug use and hepatitis C maybe additional risk factors.

## 1 Introduction

Candida Dubliniensis was first described in 1995 in a study that assessed atypical oral candida isolates found in 63 individuals, 60 of which were HIV-positive. [1] Since then, C. Dubliniensis has been recognized as an opportunistic pathogen in corneal, gastrointestinal, and urogenital compartments, as well as the blood and soft tissue. [2] Colonization of the oropharynx and respiratory tract with C. Dubliniensis has also been described in immunocompromised patients. As an invasive disease, C. Dubliniensis can also cause chronic meningitis or persistent meningeal inflammation lasting greater than a month. [3,4] However, C. Dubliniensis meningitis is exceptionally rare in immunocompetent individuals with poorly studied risk factors for colonization. [5] We present the fifth case of chronic meningitis due to C. Dubliniensis in a young, immunocompetent host, and suggest extracorporeal membrane oxygenation (ECMO) may be a risk factor for C. Dubliniensis colonization.

## 2 Case presentation

A 22-year-old immunocompetent female with a past medical history of chronic migraine headaches and polycystic ovary syndrome (PCOS) was transferred with the diagnosis of bacterial meningitis. Two years prior, she was intubated and received 1 month of ECMO for Covid-19 pneumonia with severe respiratory failure complicated by viral myocarditis & Takutsobo cardiomyopathy. Following discharge, she reported severe intractable headaches of increasing intensity, all refractory to treatments by multiple neurologists, and eventually reaching 10/10 in severity with episodes of syncope. She was evaluated by a cardiologist for syncopal episodes and was found to be bradycardic with no pathological etiology explaining her presentation. Pacemaker placement was scheduled but did not happen.

She presented for evaluation at a different institution following an episode of syncope 4 months prior to her current presentation, where she was treated with a migraine cocktail consisting of Valproic Acid, Prochlorperazine, Steroids and Magnesium Sulfate with no relief. Following discharge, she described worsening headaches and an episode of aphasia and stiffening of her extremities with retained awareness that was witnessed by her father. She denied any fevers, chills, or night sweats.

Upon admission to another institution, she underwent Magnetic resonance angiography (MRA) and Magnetic Resonance Venography (MRV), both of which did not reveal any abnormalities. Magnetic resonance imaging (MRI) of the brain revealed communicating hydrocephalus. On neurological exam, the patient demonstrated a cranial nerve VI palsy with elevated intracranial pressure, no nuchal rigidity, and negative Brudzinski's & Kernig signs. She was started on acetazolamide 250 mg two times a day. She then underwent a lumbar puncture which revealed an opening pressure of more than 55cmH20, closing pressure of 33cmH20, white blood cell count (WBC) of 166 with a neutrophilic predominance of 54%, glucose of 36, and protein level of 100. A gram stain of the cerebrospinal fluid (CSF) did not reveal any organisms, and cytology showed hypercellularity favoring a reactive/inflammatory process. There was no evidence of an abnormal or neoplastic lymphoid population. Due to concern for meningitis, she was started on empiric broad spectrum antibiotics by infectious disease consisting of Ampicillin, Ceftriaxone, Daptomycin (has severe allergy to Vancomycin), Acyclovir, and IV dexamethasone. Further analysis of CSF revealed no culture growth after 72 h, negative cryptococcal antigen, and negative polymerase chain reaction (PCR) for herpes simplex virus (HSV), pneumococcus, meningococcus, haemophilus and listeria.

**Fig. 1:**
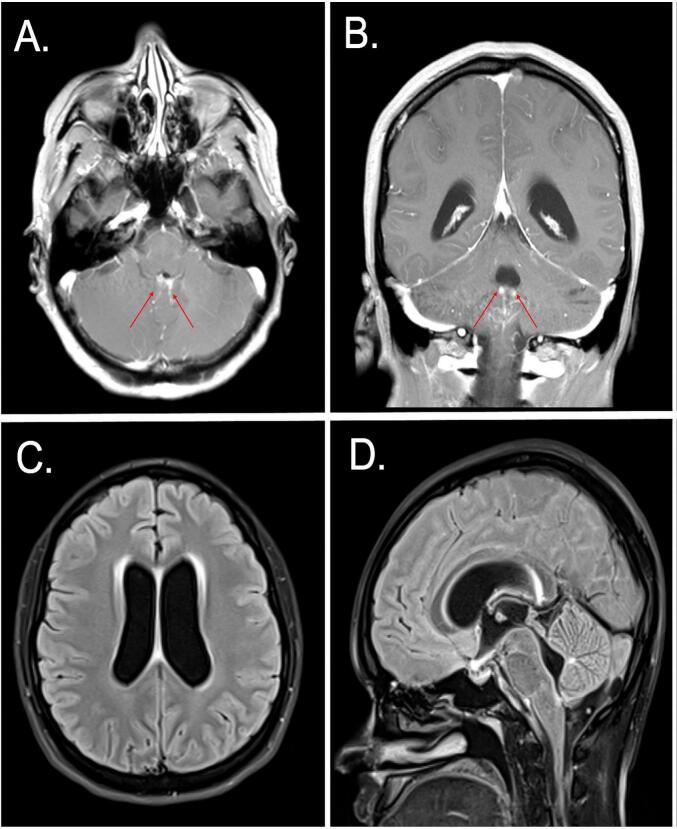
MRI of the brain revealing nodular enhancement in the brainstem (**red arrows: A, B**) and communicating hydrocephalus (**C, D**). *MRI*, magnetic resonance imaging. (For interpretation of the references to colour in this figure legend, the reader is referred to the web version of this article.)

**Fig. 2:**
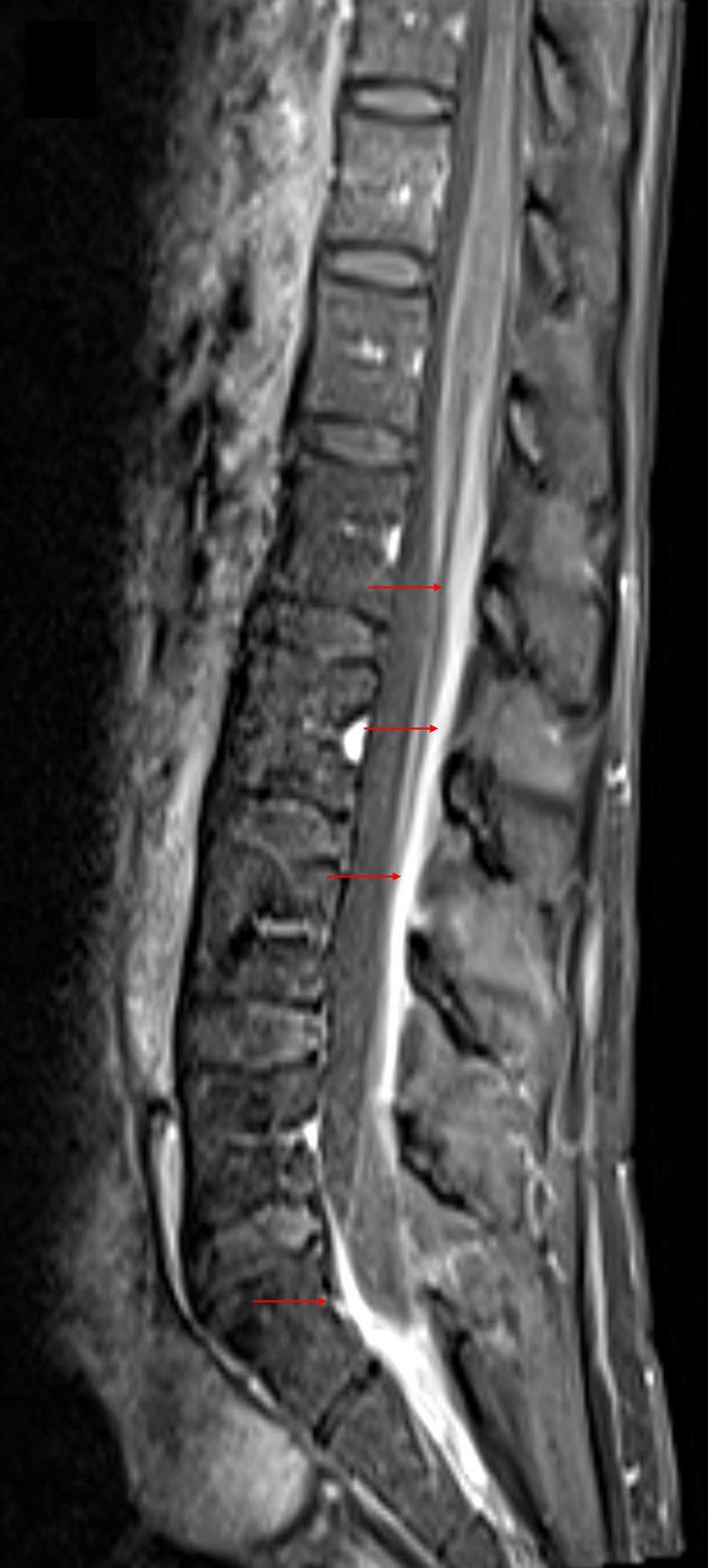
MRI of the lumbar spine revealing meningeal enhancement (**red arrows**). *MRI*, magnetic resonance imaging. (For interpretation of the references to colour in this figure legend, the reader is referred to the web version of this article.)

At current presentation at our institution, the patient was hemodynamically stable with no reported fevers. She was not taking any medications at the time, had a BMI of 20, denied alcohol, tobacco, or illicit substance use and was negative on urine drug screen, negative for HIV, and negative for hepatitis. Neurological exam was remarkable for right eye exotropia in all planes of motion and binocular horizontal diplopia that was worse on rightward gaze. No nystagmus was appreciated. Computed tomography (CT) scan of the head revealed ventriculomegaly involving lateral ventricles and temporal horns of the bilateral lateral ventricles. An MRI of the brain with and without contrast revealed prominent occipital and temporal horns, prominent peri-ependymal fluid-attenuated inversion recovery (FLAIR)/T2 signal at the level of the lateral ventricles and fourth ventricle, a degree of leptomeningeal enhancement around the brainstem, and mild papilledema/flattening of posterior globes bilaterally (Fig. 1 **&** Fig. 2). Workup for conditions with leptomeningeal enhancement including CMV, herpes, VSV, *E. coli*, West Nile virus, *Neisseria meningitidis*, streptococcus, enterovirus, and chlamydia was negative. Cytology was also negative and CD3 and CD4 counts were normal. A lumbar puncture was repeated which revealed an opening pressure of 39cmH20, closing pressure of 16cmH20, WBC of 835 with neutrophilic predominance, glucose of 4, and protein level of 134. The patient was placed on ceftriaxone, ampicillin and IV dexamethasone by infectious disease specialist. Further CSF analysis revealed negative cryptococcal antigen, negative acid-fast staining, and negative lyme screen. However, CSF cultures revealed candida Dubliniensis and a treatment regimen of Amphotericin B and Flucytosine was begun. Following multiple therapeutic lumbar punctures for increased intracranial pressure and constant headaches, the patient received a Ventriculo-Peritoneal (VP) shunt. Her headaches resolved a few days following VP shunt placement. The patient returned to baseline with resolved cranial nerve VI palsy 3 months after discharge.

## 3 Discussion

Herein, we present the fifth reported case of C. Dubliniensis chronic meningitis in an immunocompetent patient. If the onset of symptoms is considered the time of onset, the current case would represent a case of chronic meningitis of 24 months duration. The patient in this case remained afebrile throughout her admission with only one marginally elevated serum marker of inflammation. Cranial nerve palsy VI was the only neurological deficit despite the extensive leptomeningeal involvement demonstrated on MRI (Fig. 1). Diagnosis was established by repeat culture of C. Dubliniensis on 2 separate CSF samples which required 4 lumbar punctures and a large volume CSF sampling. Diagnostic approaches in the setting of CSF culture negativity include non-species-specific fungal assays such as B—D glucan [6], or next generation sequencing techniques [7]. Invasive dural tissue meningeal biopsy would provide another higher sensitivity culture avenue but would be challenging to justify in a case without features of progressive neurological decline [5].

The Infectious Disease Society of America (IDSA) treatment guidelines recommend induction therapy for candida meningitis with amphotericin B with or without flucytosine [8]. Fluconazole was selected over flucytosine for combination induction therapy in this case for several reasons. The addition of flucytosine is equivocal in terms of treatment efficacy with amphotericin B alone but with the added concerns around tolerance. In contrast, there may be synergistic benefits when combining amphotericin B and fluconazole as summarized in a recent review [9]. Although treatment failure has been reported with fluconazole alone, failure has not been reported with combined therapy. Along with potential synergistic benefits and an improved tolerance profile, this approach also allowed a smooth transition to all oral therapy with fluconazole following induction.

In contrast to the present case, all patients in the other four cases (Table 1) had a history of injecting drug use and three of the four patients had a history of hepatitis C. The patient in the present case may have contracted C. Dubliniensis when she received ECMO 2 years ago, representing another potential risk factor for C. Dubliniensis colonization.

**Table 1:** Previous cases of Candida dubliniensis in an immunocompetent host.

Characteristics	Case 1 [5]	Case 2 [7]	Case 3 [10]	Case 4 [11]	Our case
Age	25	26	27	30	22
History of injecting drug use	Yes	Yes	Yes	Yes	No
History of hepatitis C infection	Unknown	Yes	Yes	Yes	No
History of ECMO	No	No	No	No	Yes
Immunocompromised	No	Unknown	No	No	No
Duration of symptoms prior to diagnosis (months)	12	12	10	32	24
Systemic features of infection	Absent	Unknown	Weight loss	Absent	Absent
Leptomeningeal enhancement on MRI	Yes	Yes	Yes	Yes	Yes
Neurological Deficit	Radiculopathy	Saddle anaesthesia and unilateral foot drop	Complete monocular loss of vision	Mild peripheral visual field loss	Cranial nerve VI palsy
Method of microbiological diagnosis	CSF fungal Culture + dural tissue biopsy culture	CSF next generation sequencing	CSF fungal Culture	CSF fungal Culture	CSF fungal Culture

Table adapted from [11]

*ECMO*, extracorporeal membrane oxygenation; *MRI,* magnetic resonance imaging; *CSF*, cerebrospinal fluid.

Considerable points to note from this case are the striking chronicity with relatively mild but progressive symptoms, absence of systemic features, lack of spontaneous resolution, and significant diagnostic challenge. In addition, this case presents ECMO as an additional potential risk factor for C. Dubliniensis colonization along with the previously described potential risk factors of injecting drug use and hepatitis C. A diagnosis of C. Dubliniensis should be considered in patients with evidence of chronic meningitis who are immunocompetent and systemically well.

## 4 Conclusion

C. Dubliniensis is a predominantly opportunistic pathogen, typically associated with HIV-infected individuals, that can cause invasive disease in an immunocompromised host. The diagnosis of candida as a cause of chronic meningitis is often challenging and may be delayed by negative CSF culture finings. When clinical suspicion for fungal meningitis is high, repeat lumbar puncture and CSF analysis is indicated to establish a definitive diagnosis and begin treatment empirically. Additional studies are needed to confirm risk factors, like ECMO, injecting drug use, and hepatitis C, for the colonization of C. Dubliniensis, which likely predisposes individuals to invasive candidiasis. The diagnostic challenge in this case may explain the limited number of cases in immunocompetent patients within the literature.

## CRediT authorship contribution statement

**Denis Babici:** Writing – review & editing, Writing – original draft, Visualization, Validation, Supervision, Project administration, Methodology, Investigation, Conceptualization. **Ali A. Mohamed:** Writing – review & editing, Writing – original draft, Visualization, Methodology, Investigation. **Olivia Mattner:** Writing – review & editing, Validation, Investigation, Conceptualization. **Jessica Canosa:** Writing – review & editing, Validation, Investigation, Conceptualization. **Willy Gan:** Writing – review & editing, Validation, Investigation, Conceptualization. **Pooja Patel:** Writing – review & editing, Visualization, Validation, Supervision, Project administration, Methodology, Investigation, Conceptualization.

## Declaration of competing interest

This research did not receive any specific grant from funding agencies in the public, commercial, or not-for-profit sectors.
